# Valorisation of Tuff and Brick Wastes by Alkali Activation for Historical Building Remediation

**DOI:** 10.3390/ma16206619

**Published:** 2023-10-10

**Authors:** Ilaria Capasso, Gigliola D’Angelo, Marina Fumo, Mercedes del Rio Merino, Domenico Caputo, Barbara Liguori

**Affiliations:** 1 Department of Engineering and Geology, University of Chieti-Pescara “G d’Annunzio”, Viale Pindaro 42, 65122 Pescara, Italy; 2 Department of Civil, Building and Environmental Engineering (DICEA), University of Naples Federico II, P.le Tecchio 80, 80125 Naples, Italy; 3 Grupo de Investigación TEMA, Escuela Técnica Superior de Edificación, Universidad Politécnica de Madrid, 28040 Madrid, Spain; 4 ACLabs—Applied Chemistry Labs, Department of Chemical, Materials and Industrial Production Engineering, University of Naples Federico II, P.le Tecchio 80, 80125 Naples, Italy

**Keywords:** waste recycling, brick waste, geopolymers, tuff waste, alkali activation, historical building restoration, circular materials

## Abstract

Nowadays, the preservation and restoration of a historical building needs to be faced in accordance with a novel sensibility regarding the environment in order to preserve the building for future generations. In this context, the scientific community is focusing on novel and sustainable materials and techniques that allow for durability and mechanical performance as well as compatibility with the existing heritage. Alkali-activated materials represent a great challenge to the production of new materials, starting from the existing ones, with the goal of reducing consumption, emission of greenhouse gases and environmental impact. This study deals with the valorisation of waste materials coming from demolition and construction activities in the manufacture of geocomposites suitable for the restoration and conservation of historical heritage. In particular, waste from tuff sawing and brick grinding were used as raw materials, and then the geopolymeric samples produced were characterized based on a physical-chemical and mechanical point of view in order to investigate their performance and evaluate their suitability as materials for a historical building’s recovery. The results showed that brick waste-based geocomposites were more compact than the tuff-based ones, as shown by the higher-density values and the lower values of open porosity and water absorption and as further confirmed by the trend of the mechanical performance. Moreover, experimental data showed that the physical and mechanical properties of both bricks and tuff waste-based geocomposites, even with different waste content, are compatible with existing building materials as well as traditional repairing products.

## 1. Introduction

When considering interventions regarding cultural heritage or historical built heritage, it is fundamental to investigate, in a transversal way, the themes inherent to traditional materials and techniques and their degradation. At the same time, the innovative approach of modern techniques and materials, which respond in a global way both to the compatibility problems and to the growing environmental emergencies that characterise today’s daily life, has to be seriously considered.

In fact, it is important to emphasise that, in addition to the problems created by the huge quantities of waste material produced [1], in Italy, most of the building stock is not only extremely poorly maintained [2] but is also characterised by a historical building that, although not subject to constraints, requires all of the measures related to the compatibility of materials that are typical of the sensitivity adopted in the field of restoration.

The Cultural Heritage and Landscape Code defines restoration (in Art. 29, paragraph 4) as: “the direct intervention on the property through a complex of operations aimed at the material integrity and recovery of the property itself, at the protection and transmission of its cultural values”. The interaction with the environment, in fact, entails that over time an alteration of the original characteristics of the artefacts and the materials that compose them will occur; they will undergo degradation phenomena due to physical agents such as humidity, water, thermal shock or even wind erosion or photo-oxidative phenomena caused by solar radiation, biological agents and chemical agents. The study of materials and techniques to be used for the recovery of this ‘material integrity’ must also be guaranteed for that part of the unrestricted historical heritage that needs to be recovered. Moreover, reuse of materials as a recovery practice has its origins in a very ancient time where the practice of spoliation was very common and abandoned building heritage, both residential and monumental, was literally reused as a quarry of materials to be utilized in the construction of new buildings [3].

Actually, the valorisation of construction and demolition waste (CDW) materials represents a kind of return to the origins, suitable to solve the problem of the disposal of huge quantities of waste and, at the same time, guarantee compatibility with the historical substrate using innovative mixtures that reproduce the same mineralogical and physical features [4,5,6,7]. Accordingly, geopolymers and alkali-activated materials represent the most promising and sustainable alternative to cement-based binders/concretes [8,9,10,11,12]. Geopolymers are obtained by chemically reacting a starting alumino-silicate powder with a strongly concentrated aqueous alkali hydroxide and/or silicate solution, resulting in the production of a synthetic amorphous-to-semicrystalline alkali alumino-silicate new phase [13,14,15]. In the last decade, the production of geopolymeric materials from industrial waste, agricultural waste and municipal waste has been widely explored [16,17,18,19,20,21,22,23,24,25,26,27]. In fact, waste-based geopolymers offer several advantages which contribute to environmental sustainability and economic benefits. The use of various kinds of solid waste can lead both to different performances and properties of geopolymers, depending on the waste’s own features, such as chemical composition, morphology, particle size, and water absorption as well as to their different ways of being used, such as precursors, aggregates, fibres, etc. Waste-derived geopolymers combine all the excellent features typically related to geopolymeric materials (high durability, thermal and fire resistance, resistance to chemical corrosion, high mechanical strength) [11,13] with high sustainability, reduction in waste disposal, conservation of natural resources and potential to lower CO_2_ emissions. For these reasons, scientific research is always more interested in finding new ways to optimize the use of waste geopolymers for various innovative applications and to address the challenges related with their use. In particular, considering the chemical nature of construction and demolition (C&D) waste, geopolymerization technology has begun to be considered as an advantageous and smart reusable possibility for them instead of landfilling [28,29,30,31]. The use of alkali-activated material in the building sector can provide a low environmental impact either in terms of energy consumption or natural resource saving, together with high mechanical and durability performances [11]. Furthermore, in regard to historical building restoration, chemical, physical and mechanical compatibility with the original material becomes mandatory, as well as the ability to show similar aesthetic features [32,33,34,35,36]. Chemical-physical and mechanical properties, combined with other specific peculiarities of geopolymeric materials, such as fast drying and good adhesion to the ceramic, allow their potential use as an alternative to traditional materials in the conservation and restoration of cultural heritage [33,37,38,39,40,41]. Starting from these considerations, this paper reports the progress of the research experiments carried out on the production of waste-based geocomposites, which were produced from waste deriving from the demolition and construction activities of historical heritage, in order to promote the possible future applications of innovative and sustainable materials in restoration and conservation activities.

## 2. Experimental

Details of geopolymer precursors, additives and alkaline activator solutions used for the production of geocomposites are provided.

### 2.1. Raw Materials and Preparation of the Geocomposites

**Figure 1:**
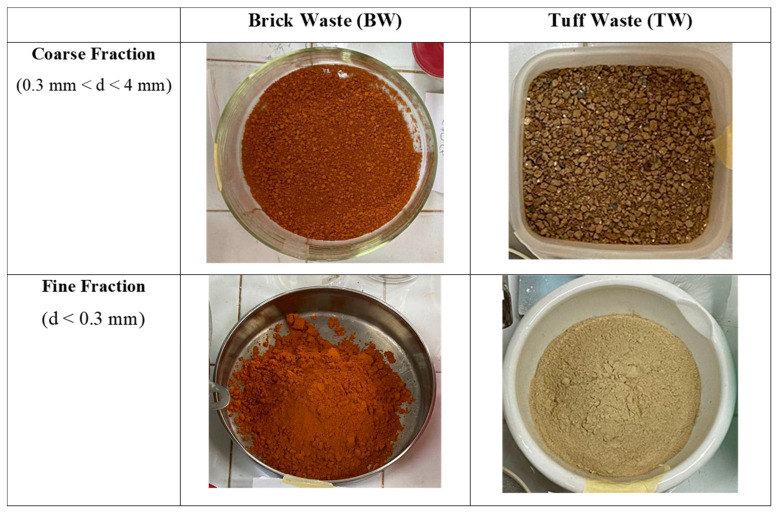
Raw materials used for the production of the geopolymeric samples (d = particle size).

Starting with the identification of local resources, strongly integrated with the territory from a historical point of view, different types of construction and demolition waste were used to produce geocomposites either as geopolymer precursors or as natural aggregate replacements (Figure 1):

(1) Waste from red clay bricks (BW): a commercial product supplied by CTS s.r.l. (Altavilla Vicentina, Italy) and recovered from construction and demolition activities, properly sieved and divided it into two particle-size fractions of less than 0.3 mm and between 0.3 and 4 mm.
(2) Waste of tuff sawing (TW): a mixture of two typical Italian tuffs, such as Neapolitan Yellow Tuff (NYT) and Viterbo red tuff (VT), was used. TW was crushed and sieved to select two particle-sized fractions of less than 0.3 mm and between 0.3 and 4 mm.
(3) Fly ashes (FA): derived from combustion in coal-fired power plants for the production of electricity; they were also added as partial replacement of the waste (10 or 20%).

A sodium silicate solution (SS) (Na_2_O 8.15%, SiO_2_ 27.40%), provided by Prochin Italia S.r.L. (Caserta, Italy), and a 10 M sodium hydroxide solution (N), prepared by dissolving NaOH in pellets (NaOH 98%, J.T. Baker) in bi-distilled water, were used as activators.

Geocomposites were produced as follows: powdered materials were previously dry-mixed and homogenized, and then the activator solution was added to the dry mixture. An aggregate/binder ratio of 0.5 was selected.

**Figure 2:**
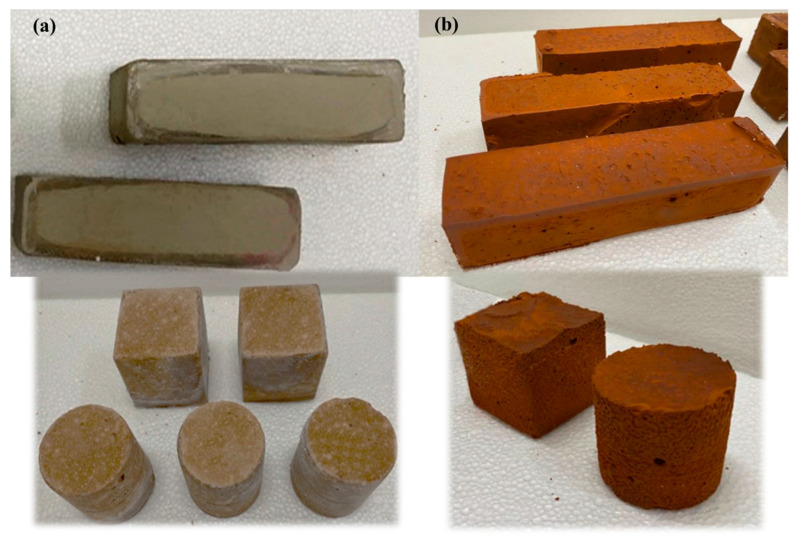
TW-based (**a**) and BW-based (**b**) geocomposite specimens produced.

The alkaline activator solution was prepared by mixing sodium silicate solution (SS) with 10 M sodium hydroxide solution (N). The weight ratio SS/N/binder was 1:1:3. The activator/binder ratio was fixed at 0.66 for all the mixtures. Both the experimental parameters and the procedure used to manufacture the geocomposites were properly selected and optimized from previous studies [16,28]. For each waste, three mixtures were prepared using 0, 10 or 20% of fly ashes as partial replacement. Specimens were prepared in three different geometries according to the specific requirements of the European standards for physical and mechanical characterization (see Figure 2):

- cylindrical (diameter 45 mm; height 50 mm),
- cubic (side 50 mm),
- prismatic (40 × 40 × 160 mm^3^).

The two different granulometry fractions were used as follows: fine fraction, less than 0.3 mm, as binder precursor and coarse fraction, with particle size between 0.3 and 4 mm, as aggregate.

The curing conditions selected for all the specimens were: 3 days, 60 °C in an oven, 100% relative humidity (sealed vessels). At the end of the curing, all the specimens were removed from the moulds and stored at room temperature for 28 days, following the prescription for traditional cementitious materials in terms of samples ageing, in order to perform the whole characterization in standard and comparable conditions. All the tests were performed in triplicate.

The compositions, the labels of all the manufactured geocomposites and the main experimental parameters are summarized in Table 1.

### 2.2. Mineralogical and Chemical Characterization of Raw Materials

The mineralogical composition was evaluated by XRD analysis on a powder sample using a Panalytical X’Pert Pro diffractometer equipped with a PixCel 1D detector (operative conditions: CuKa1/Ka2 radiation, 40 kV, 40 mA, 2θ range from 5 to 80, step size 0.0131 2θ, counting time 40 s per step). X-ray fluorescence spectroscopy (XRF; AXIOS Panalytical Instrument; Malvern PANalytical, Almelo, The Netherlands) has been performed to determine the chemical composition of samples in the form of pressed pellets.

### 2.3. Physical and Chemical Characterization of the Geopolymeric Mortars

The water absorption under vacuum and the open porosity of the geocomposites were measured according to the Italian Standard UNI 11060 [42]. Firstly, the specimens were dried at 60 ± 5 °C until constant mass (*M*_1_, g) was reached. Then, they were immersed in water at room temperature in an evacuation vessel and the pressure was lowered to about 20 mmHg and kept constant for 2 h. After that, pressure was returned to atmospheric value and the samples were first weighed immersed in water (hydrostatic weighing, *M*_2_, g) and finally, after being gently wiped with a damp cloth, they were weighed again, determining their water-saturated mass (*M*_3_, g). Each test was performed in triplicate and the results are the average values.

The water absorption (*WA*%) and the open porosity (*OP*%) were expressed as follows:

$$WA\%=\frac{M_{3}-M_{1}}{M_{1}}\times 100$$

$$OP\%=\frac{M_{3}-M_{1}}{M_{3}-M_{2}}\times 100$$

The values of apparent and real density (δ_A_ and δ_R_, respectively) of the geopolymeric samples can be also deduced from the measurements performed according to the Italian Standard [42]. Moreover, capillarity tests were carried out in accordance with European Standard UNI EN 15801 [43] in order to assess the amount of water absorbed (Q) per surface unit as a function of time. Tests were performed in triplicate and then the mean value of capillary absorption coefficient (CA, g·cm^−2^·s^−1/2^) was determined. Considering that, for short times, the relation between the water adsorbed (Q) and the square root of time is quite linear, CA value may be evaluated as the slope of the straight line in the first 30 min of the capillarity test [43]. Finally, the degree of geopolymerization and the influence of the waste addition on the chemical features of the produced samples were deduced by means of FTIR spectroscopy, performed at room temperature by using a Spectra 3000 (Perkin Helmer, Waltham, MA, USA) in ATR mode, and selecting a wavenumber resolution of 4 cm^−1^ for 32 scans from 4000 to 600 cm^−1^.

### 2.4. Mechanical Characterization of the Geopolymeric Mortars

The flexural strength tests were performed according to UNI EN 196-1:2016 [44] on prismatic specimens with dimensions 40 × 40 × 160 mm^3^, using the Ibertest as the testing machine. For the compressive strength tests, the load is applied to the two broken portions of the specimens from the previous flexural strength tests. All the tests were carried out after a curing time of 28 days. Further information about the mechanical properties has been obtained by a surface hardness test [45], considering the hole that is produced on the sample under test, following the application of a fixed force on each sample, measured in Shore D units varying in a range from 0 (softest) to 100 (hardest).

## 3. Results and Discussion

### 3.1. Characterization of the Raw Materials

**Figure 3:**
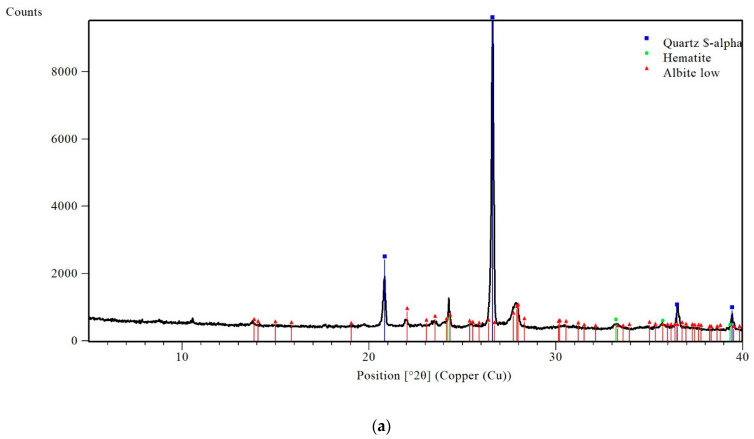

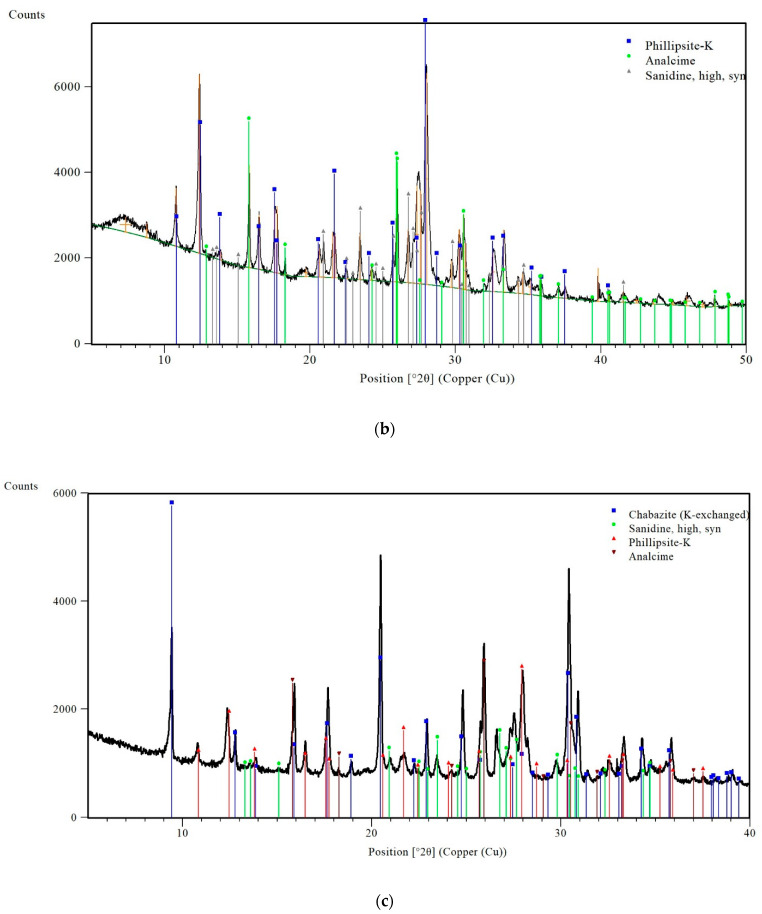

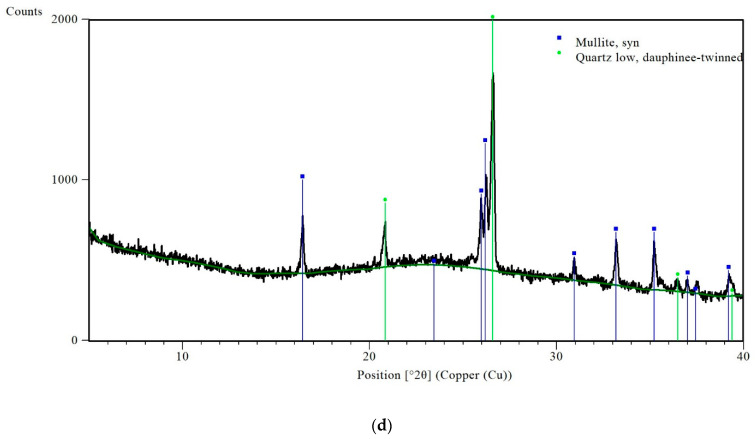
XRD spectra of the raw materials: (**a**) BW, (**b**) NYT, (**c**) VT and (**d**) FA.

Crystalline structures of the geopolymer precursors, which were analysed with X-ray diffraction (XRD) technique, are shown in Figure 3.

Chemical compositions of the raw materials, used for the production of geopolymeric samples and reported in Table 2, confirmed the main silico-aluminate nature of all of them. The oxides of the major elements determined were SiO_2_, TiO_2_, Al_2_O_3_, Fe_2_O_3_, MgO, CaO, Na_2_O and K_2_O, whose concentrations are expressed in weight percentages (wt.%).

### 3.2. Physical and Chemical Characterization of the Geopolymeric Mortars

The effects of waste addition on some relevant physical characteristics of the geopolymeric composites (i.e., density, open porosity and water absorption) were evaluated and the experimental results, as average values of triplicate tests, are reported in Table 3. The apparent density values of both TW-based and BW-based geopolymeric mortars remain basically unchanged regardless of the waste content. It is worth noting that the BW-based geocomposites showed higher density values compared to the TW-based ones, revealing a higher compactness, further confirmed by the lower values of open porosity and water absorption, as well as the experimental results presented in the following sections. Moreover, considering the effects of waste addition on the open porosity of all the geopolymeric samples, it is possible to deduce that samples produced without FA as partial replacement turned out to be the most porous ones. This can be explained by considering that FA, even in low percentages, promoted the formation of more compact geopolymeric matrices [16,48], as confirmed also by the lower values of water absorption of all the samples containing FA. In particular, this effect is more evident for the BW-based samples, which showed very similar values of open porosity and water absorption regardless of the specific amount (10 or 20%) of FA added and lower if compared to BW100 sample. On the contrary, the open porosity and water absorption of TW-based geocomposites were affected by the percentages of FA added, exhibiting increasing values with decreasing FA amount. This could be explained by taking into account the lower reactivity of tuff waste powders, due to the higher crystallinity of their silico-aluminate species, which caused the TW-based samples to be more affected by the presence of FA.

**Figure 4:**
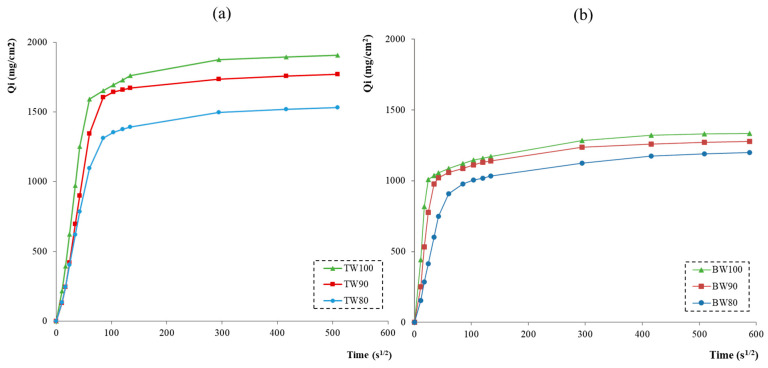
Average values of capillary water absorption (Q) in function of time for (**a**) TW-based and (**b**) BW-based geocomposites.

The capillary absorption curves and the relative values of capillary absorption coefficients for all geocomposites are reported, respectively, in Figure 4 and in Table 3. The data indicated that all the samples reached water saturation after 48 h. Moreover, the water absorption rate decreased with time, probably as consequence of the increased water content inside the specimen and of the reduced participation of the less-accessible pores [49]. At the same time, it is possible to note that the absorption rate, especially in the first minutes of the test, increased with the increase of the waste amount added, following the same trend of the porosity values (Table 3). Capillary absorption coefficients showed higher values for the geopolymeric samples produced without FA addition, as expected from the porosity results (see Table 3). Finally, considering the values of Q_max_ reported in Table 3, it is worth noting that, in accordance with previous results, BW-based geopolymers showed lower amounts of absorbed water by capillarity, further confirming their higher compactness.

**Figure 5:**
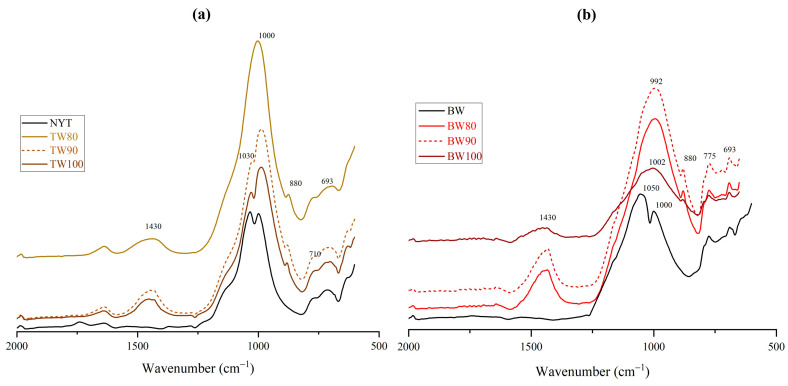
FTIR spectra for (**a**) NYT and TW-based geopolymers and for (**b**) BW and BW-based geopolymers.

The analysis of FTIR spectra is a valid method of investigating and monitoring the chemical modification of the silico-aluminate phases involved during the geopolymerization process [50]. The FTIR spectra of waste-based geopolymers (Figure 5a,b) turned out to be characterized by a broad absorbance band, ranging between around 700 and 1200 cm^−1^, typical of the overlapping of single peaks related to the asymmetric stretching of T–O–Si (T = Si and Al) bonds present in the amorphous structure of aluminosilicate [51]. Moreover, all the geopolymeric systems showed absorbance at around 1430 cm^−1^ and at 880 cm^−1^ attributed to the stretching of carbonate ions in sodium carbonate [52]. In the FTIR spectra of NYT and TW-based samples (Figure 5a), the symmetric stretching vibration of Si–O and Al–O was noticed in the range of 750–690 cm^−1^ and attributed to the presence of phillipsite [53]. The peaks appearing at about 780 cm^−1^ and at 693 cm^−1^ in Figure 5b are assigned to quartz as the crystalline phase in the starting brick waste powder. The comparison between the spectra of raw materials (black lines in Figure 5a,b) and geocomposites was evidenced by a slight shift of the T–O–Si asymmetric stretching band towards lower wavenumbers, in particular from region about 1030–1000 cm^−1^, typical of zeolites, for NYT [53] and from around 1050 cm^−1^ for BW. This spectral change can be related to chemical modifications of the aluminosilicate structure of the raw materials induced by the alkaline activation [54].

### 3.3. Mechanical Characterization of the Geopolymeric Mortars

The average values of flexural and compressive strength for each geopolymeric mixtures are reported in Table 4.

The mechanical properties followed the same trend already evident for the physical properties. In fact, the addition of FA, regardless of the 10 or 20%, led to more resistant geopolymeric matrices, in terms of both flexural and compressive behaviour, for both TW-based and BW-based specimens, as expected also from porosity values (Table 3). Moreover, it is worth noting that BW-based geocomposites showed much better mechanical performance with a compressive strength equal to ≈13 MPa for BW80 and BW90 samples, which is much higher if compared to that equal to ≈1.5 MPa of the corresponding TW-based geopolymeric samples. Probably, the improvement of compressive strength for BW-based samples can be related to a more effective leaching of silica and alumina from the brick waste powder at high alkalinity, which promoted the geopolymerization process [55]. Furthermore, mechanical properties of geocomposites depend not only on the strength of the geopolymeric binder, but also on the mechanical response of aggregates and on the interface between binder and aggregate produced during the consolidation process [16]; so in this case, the adherence at the interface developed by tuff powder, used as aggregate, was worse than the brick waste.

The results obtained from the Shore-D surface hardness tests are shown in Table 5 and further confirmed the tendency of the mechanical properties previously discussed. In fact, as expected, all the BW-based geocomposites exhibited higher hardness values, in particular, the BW80 and BW90 samples which showed very similar hardness in accordance with density values (see Table 3).

**Figure 6:**
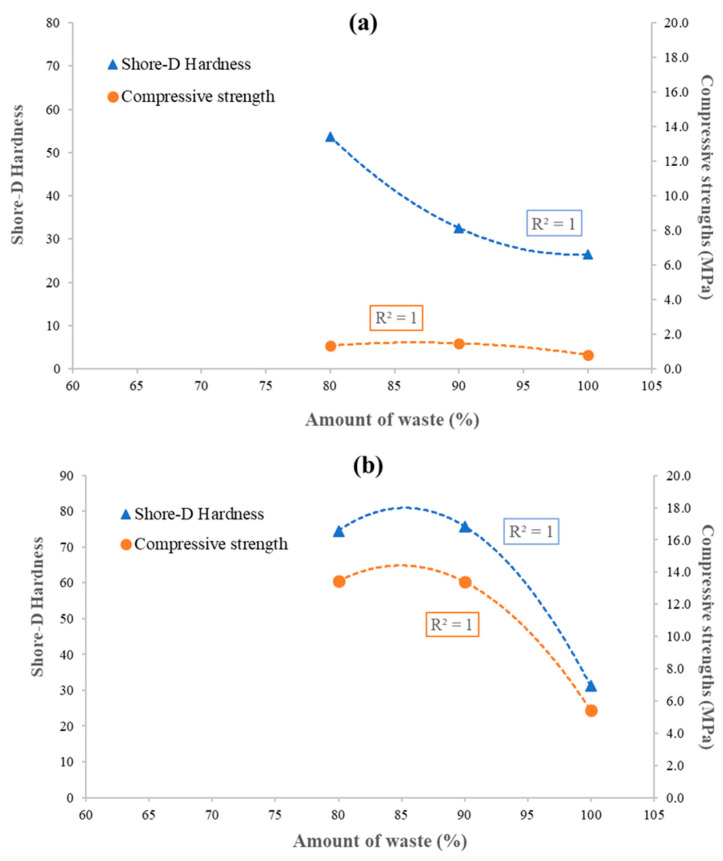
Correlation between amount of waste, Shore-D hardness and compressive strength for (**a**) TW-based and (**b**) BW-based geocomposites.

The correlations of the waste amount with shore-D hardness and compressive strength values for TW-based and BW-based geocomposites are reported in Figure 6. For all the typologies of waste-based geopolymers, correlation was well described by the second-degree polynomial function with a correlation coefficient equal to R^2^ = 1 for both shore-D hardness and compressive strength. This means that the increase in waste amount causes a decrease in mechanical properties, following a fluctuating trend.

**Figure 7:**
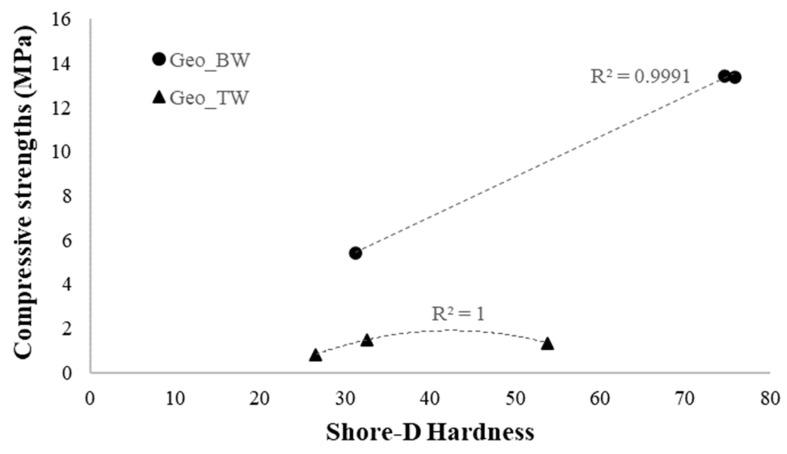
Correlation Shore-D hardness and compressive strength for TW-based and BW-based geocomposites.

Figure 7 shows the relationship between compressive strength and Shore-D hardness of all the geocomposites. For the BW-based geopolymers, compressive strengths are linearly correlated to the hardness value, with a correlation coefficient R^2^ ≈ 1, while the data of the TW-based samples do not follow a linear relationship, but a second-degree polynomial function (R^2^ = 1). This suggests that the typical methods of using hardness testing to estimate the strength may not be applicable to TW-based geocomposites.

### 3.4. Comparison with Typical Materials Used in Cultural Heritage

To validate and verify the suitability of TW-based and BW-based geocomposites in the restoration and conservation of cultural heritage, some of the experimental results obtained in this paper were compared with the scientific literature for several typologies of traditional and widely used products. Historical mortars, in fact, are very complex systems, containing aerial or hydraulic binders or a blend of them, aggregates and eventually additions. For this reason, different traditional mortars made with aerial lime and natural hydraulic lime (NHL) were selected as the basis for comparison (see Table 6). Moreover, considering that, in order to design compatible restoration mortars, it is fundamental to investigate the physical and mechanical properties of both the original and repair mortars, an experimental repair mortar, a natural limestone and a cement-based mortar were also considered as reference materials for comparison with the geopolymeric samples.

Porosity, capillary absorption and mechanical properties have been selected as the main factors used to verify the compatibility between ancient and restored structures [56]. As regards physical properties, it is possible to determine that the BW-based samples showed similar values of open porosity and capillary absorption coefficient, especially the BW80 and BW90 samples (see Table 3), and even lower amounts of water absorbed by capillarity, if compared to all the reference mortars (Table 6). TW-based geocomposites exhibited higher open porosity and capillary absorption coefficients (Table 3) but were always comparable to existing building materials [57].

Taking into account that repair mortars should have similar behaviour in the presence of water, especially in relation to their permeability to water and to water vapour compared to the existing masonry materials, the geocomposites’ performance can be considered more than adequate in terms of compatibility because their physical features should allow a quick water evaporation through the mortar pores, which is also a key factor in salt-induced decay. Moreover, a repair mortar should not be stronger than the existing one and, at the same time, not be weaker than the masonry units [58]. Accordingly, TW-based samples showed compressive strengths very similar to the reference mortars. BW-based geopolymers were characterized by higher values of flexural and compressive strength (Table 3 and Table 6), in accordance with the requirements for historical mortars.

**Table 6:** Physical and mechanical properties of some mortars widely used for restoration purposes.

Sample	Open Porosity (%)	CA (mg/cm^2^ s^−1/2^)	Q_max_ (mg/cm^2^)	Compressive Strength (MPa)	Flexural Strength (MPa)
Aerial lime mortar CL-90 [59,60]	25.90	13.90	2870	0.48	0.24
Hydraulic lime mortar NHL 5 [59,60]	23.50	9.40	3970	3.70	1.00
OPC mortar (CEM II B/L 32.5) [60]	18.80	3.40	2030	24.80	4.70
Repair mortar NHL-Z 3.5 with crushed bricks and silica sand [56]	26.23 *	24.16 *	n. d.	3.48	n. d.
Historical magnesian mortar [56]	30–40	28.51	n. d.	n. d.	n. d.
Natural Bioclastic Limestone [56]	24–30	31.32	n. d.	10.09	n. d.

* Evaluated after 12 months. n. d.: not determined.

## 4. Conclusions

Working on the heritage means not only preserving its historical, cultural and landscape value, but also promoting a unique economic resource, promoting social development and increasing environmental protection. The direction to follow must therefore aim at the approach of circular economies, considering conservation not only as a limitation, but as a means of re-functionalizing and redeveloping spaces in an innovative way without forgetting or erasing the intrinsic value of the territory.

Bricks and tuff wastes coming from construction and demolition activities have been successfully used to produce geocomposites suitable to restoration practices. A deepened physical-chemical and mechanical characterization of the geopolymeric samples was carried out order to investigate their performance. In particular, brick waste-based samples showed higher density and lower values of open porosity and water absorption than the tuff-based ones. The higher compactness of the brick waste-based geocomposites was further confirmed by their mechanical performance. Finally, experimental results showed that the physical and mechanical properties of both brick and tuff waste-based geocomposites, also with different waste content, are compatible with existing building materials and with all the traditional repairing products.

Accordingly, the alkali activation of construction waste can represent a novel and sustainable approach that promotes performance and compatibility achievement in the protection and restoration of heritage buildings.

## Figures and Tables

**Table 1:** Geocomposite labels, relative compositions and experimental parameters.

Sample	Binder (Fine Fraction, %wt)			Ag./Bin. Ratio	Act. Sol.	NaOH	Liquid/Powder	Curing Time	Curing Temperature
	FA	BW	TW						
BW80	20	80	/	0.5	NaOH + SS	10 M	0.6	72 h	60 °C
BW90	10	90	/						
BW100	/	100	/						
TW80	20	/	80						
TW90	10	/	90						
TW100	/	/	100						

**Table 2:** Chemical compositions of the raw materials.

Sample	Major Elements (wt%)							
	SiO_2_	Al_2_O_3_	Fe_2_O_3_	MgO	Na_2_O	K_2_O	CaO	TiO_2_
BW [28]	47.90	31.82	2.99	4.14	3.75	3.59	4.52	/
NYT [46]	58.82	19.10	4.60	1.11	3.44	9.39	3.10	0.53
VT [47]	53.75	16.86	4.00	1.55	1.60	8.15	5.21	0.51
FA	58.13	23.28	5.98	2.04	0.97	2.80	4.22	1.02

**Table 3:** Main physical properties of geopolymeric mortars.

Sample	Apparent Density (g/cm^3^)	Open Porosity (%)	Water Absorption (%)	CA (mg/cm^2^ s^−1/2^)	Q_max_ (mg/cm^2^)
TW80	1.35 ± 0.08	39.44 ± 1.03	26.70 ± 0.97	20.97 ± 0.34	1518
TW90	1.40 ± 0.01	41.92 ± 0.96	29.89 ± 0.87	23.93 ± 1.19	1770
TW100	1.37 ± 0.01	42.32 ± 0.54	30.97 ± 0.48	33.36 ± 0.11	1907
BW80	1.76 ± 0.01	27.68 ± 0.50	15.71 ± 0.38	19.53 ± 0.82	1137
BW90	1.75 ± 0.01	26.24 ± 0.01	15.02 ± 0.04	21.89 ± 0.24	1236
BW100	1.73 ± 0.01	34.16 ± 0.21	19.75 ± 0.02	26.67 ± 1.89	1333

**Table 4:** Flexural and compressive strengths of the geocomposites.

Sample	Flexural Strength (MPa)	Compressive Strength (MPa)
TW80	1.23 ± 0.05	1.34 ± 0.18
TW90	0.62 ± 0.04	1.48 ± 0.08
TW100	0.39 ± 0.01	0.81 ± 0.06
BW80	4.58 ± 0.06	13.44 ± 0.36
BW90	3.60 ± 0.30	13.39 ± 0.47
BW100 *	2.85 ± 0.73	5.34 ± 0.66

* Data already reported in [28].

**Table 5:** Shore-D hardness test results.

Sample	Surface Hardness
TW80	53.73 ± 2.20
TW90	32.56 ± 2.32
TW100	26.50 ± 0.82
BW80	74.55 ± 1.17
BW90	75.80 ± 3.02
BW100	31.17 ± 0.33

## Data Availability

Data is contained within the article.
